# Impact of quadrivalent influenza vaccines in Brazil: a cost-effectiveness analysis using an influenza transmission model

**DOI:** 10.1186/s12889-020-09409-7

**Published:** 2020-09-09

**Authors:** Pascal Crépey, Louis Boiron, Rafael Rodrigo Araujo, Juan Guillermo Lopez, Audrey Petitjean, Expedito José de Albuquerque Luna

**Affiliations:** 1 Department of Quantitative Methods in Public Health, UPRES-EA-7449 Reperes, EHESP, University of Rennes, 15 Av. Professeur Léon Bernard, 35043 Rennes, France; 2 Sanofi Pasteur, Av. das Nações Unidas, 14410 - Condomínio Parque da Cidade Torre Sucupira, Jardim Morumbi – CEP, São Paulo, SP 04794-000 Brazil; 3 Sanofi Pasteur, Av. Universidad 1738, Santa Catarina, Coyoacán, 04000 Ciudad de México, CDMX, Mexico; 4 Sanofi Pasteur, 14 Espace Henry Vallée, 69007 Lyon, France; 5 Institute of Tropical Medicine (IMT) - University of São Paulo (USP), Av. Dr. Enéas Carvalho de Aguiar, 470 - Jardim America – CEP, São Paulo, SP 05403-000 Brazil

**Keywords:** QIV, Quadrivalent, Public health, Cost-effectiveness, Vaccine, Influenza, Brazil

## Abstract

**Background:**

Influenza epidemics significantly weight on the Brazilian healthcare system and its society. Public health authorities have progressively expanded recommendations for vaccination against influenza, particularly to the pediatric population. However, the potential mismatch between the trivalent influenza vaccine (TIV) strains and those circulating during the season remains an issue. Quadrivalent vaccines improves vaccines effectiveness by preventing any potential mismatch on influenza B lineages.

**Methods:**

We evaluate the public health and economic benefits of the switch from TIV to QIV for the pediatric influenza recommendation (6mo-5yo) by using a dynamic epidemiological model able to consider the indirect impact of vaccination. Results of the epidemiological model are then imputed in a health-economic model adapted to the Brazilian context. We perform deterministic and probabilistic sensitivity analysis to account for both epidemiological and economical sources of uncertainty.

**Results:**

Our results show that switching from TIV to QIV in the Brazilian pediatric population would prevent 406,600 symptomatic cases, 11,300 hospitalizations and almost 400 deaths by influenza season. This strategy would save 3400 life-years yearly for an incremental direct cost of R$169 million per year, down to R$86 million from a societal perspective. Incremental cost-effectiveness ratios for the switch would be R$49,700 per life-year saved and R$26,800 per quality-adjusted life-year gained from a public payer perspective, and even more cost-effective from a societal perspective. Our results are qualitatively similar in our sensitivity analysis.

**Conclusions:**

Our analysis shows that switching from TIV to QIV to protect children aged 6mo to 5yo in the Brazilian influenza epidemiological context could have a strong public health impact and represent a cost-effective strategy from a public payer perspective, and a highly cost-effective one from a societal perspective.

## Background

Seasonal epidemics of influenza are a major public health burden worldwide. Respiratory deaths alone are estimated between 290,000 and 650,000 every year worldwide [1, 2]. In Latin America and the Caribbean area, the burden of influenza-like illness (ILI) has been estimated in 2008 to be between 164 and 251 million cases [3]. In Brazil, influenza epidemics significantly weight on the Brazilian healthcare system and on society with estimation of the number of ILI cases as high as 83 millions for a single year [3]. Spatio-temporal patterns of influenza epidemics in the region display substantial heterogeneity both in terms of timing [4] and influenza virus circulation [5], hence challenging the design and efficiency of influenza vaccination campaigns [6].

Influenza vaccination is the most efficient way to prevent the disease and its consequences. In Brazil, vaccination against influenza started in 1999 targeting elderly older than 65-year-old (yo), to later extend it to elderly aged 60 and over, among other high-risk groups, such as individuals with chronic diseases, diabetes, young children and others. Public health authorities have then progressively expanded the recommendation to the pediatric age-groups by first including, in 2011, children aged from 6 months to under 2yo [7], then under 5yo in 2016 [8], and in 2019 to all children from 6 months to 6 years of age [9]. However, in the past years, influenza vaccines have been criticized for the variability of their effectiveness, partially related to mismatch between the vaccine strains annually recommended by WHO, and the influenza strains circulating during the influenza season. To mitigate this issue, new quadrivalent influenza vaccines (QIV) have been developed. They contain two influenza A strains (A/H1N1 and A/H3N2) as well as two B lineages (B Victoria and B Yamagata), while previous trivalent influenza vaccines (TIV) only contained one B lineage. They have been recommended and shown their improved effectiveness as well as their cost-effectiveness in replacing TIV in several countries [10–13]. In Brazil, the public health and economic benefits of QIV have been demonstrated using various methodologies, but all relying on a “static model” approach, hence not accounting for the indirect impact of vaccination [12, 13]. While these approaches are recommended by WHO guidelines for economic evaluation of vaccination programs targeting individuals with high risk of severe complications, they are not well suited when vaccination targets age-groups “likely to change population disease transmission substantially” [14]. Hence, the purpose of the current analysis is to evaluate the public health and economic value of switching the pediatric influenza recommendation (6mo-5yo) from TIV to QIV using a dynamic epidemiological model able to consider the indirect impact of vaccination.

## Methods

### Epidemiological model design

We adapted a dynamic transmission model used to assess the impact of QIV in the US, first published by Crépey et al. [15]. The model is a compartmental model able to simulate infections by two A subtypes or two B lineages at the same time. Individuals can be susceptible to infection (S), exposed but not infectious (E), infectious (I), or recovered (R) from an infection and therefore immune. In addition, individuals can be vaccinated (V) against both B influenza lineages, and either one of the two. The model accounts for cross-immunity against a B lineage induced by vaccine containing the opposite B lineage or induced by natural infection. In addition, the model simulates several epidemic seasons in a row in order to take into account the evolution in time of the immune status of the population. Because time period of simulations can span several years, population ageing is considered as continuous process. To better handle Brazilian immunization policies, we changed the age distribution of the original model into 8 age groups (0-5mo, 6mo-5yo, 6yo-9yo, 10yo-14yo, 15-19yo, 20-39yo, 40-59yo, and older than 60yo). Since inter-individual contacts data within Brazil are not available, we retained the inter-individual contact matrix used in the original version of the model, assuming that any differences between the US and Brazil population contact structure would have a minor impact in the scope of this study. The model was developed in R 3.5.3 [16] and C++ [17], and a full description of its set of differential equations is provided in [15].

### Economic model design

Our economic model is similar in structure to the one published by de Boer et al. [18]. It is a decision tree-based model where symptomatic individuals infected with influenza will have various probabilities of having an outpatient visit, being hospitalized, or dying from influenza, depending on their age and whether they are at-risk of severe consequences. The economic model computes health outcomes (outpatient visits, hospitalizations, and deaths), health effects (life year lost, quality adjusted life year lost), medical costs, vaccination costs and indirect costs (productivity losses). Age-stratified outputs of the epidemiological model are used as inputs of the economic model, developed in Excel© 2010.

### Epidemiological data

The proportion of influenza A and B circulating on the period 2010–2017 was extracted from the Brazilian SINAN notification system [19]. The split for A/H1N1 and A/H3N2 was obtained for Brazil either from WHO Flunet [20](2010–2012) or from SINAN (2013–2017), and the split of B lineages in Brazil from Luna et al. [21]. We obtained hospitalization data over the same period from the Brazilian public health care system (Sistema Único de Saúde, SUS) [22]. As the SUS only accounts for public hospitals, and since approximately 25% of the Brazilian population have access to private hospitals, we extrapolated the number of hospitalizations obtained from the public sector proportionally to estimate the total burden of influenza hospitalizations, assuming that the same incidence is observed in both public and private hospitals. We obtained the proportion of the Brazilian population covered by the private system over the period of analysis from the National Health Agency (ANS) [23].

**Fig. 1:**
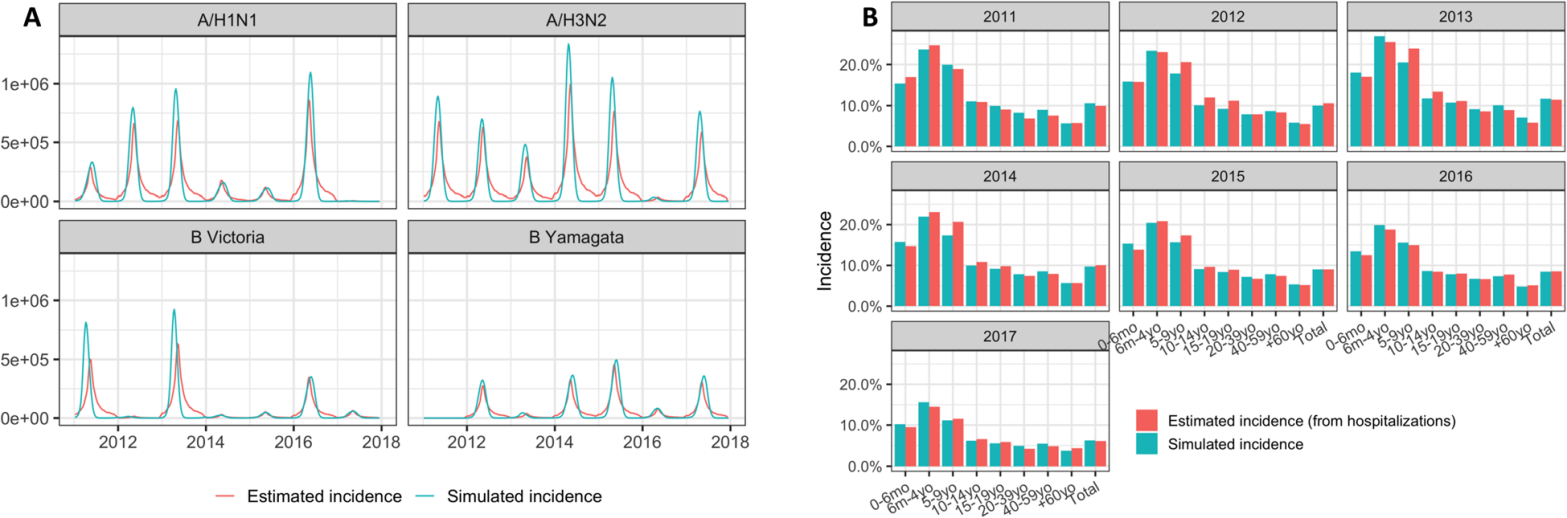
Results of the epidemiological model compared to estimated influenza incidence in Brazil (2011–2017). Plot A shows the weekly incidence by influenza subtypes and lineages over the period. Plot B show the yearly incidence by age-groups

Following the same approach used by the US CDC for influenza burden estimation [24], we divided the number of hospitalizations by the test sensitivity and by the percentage of tested subjects to account for non-tested and false negative subjects. Next, we multiplied this estimation by a case-hospitalization ratio to obtain a first estimation of the total number of influenza cases seen by the healthcare system [25]. Finally, we divided this estimation by the probability to seek for healthcare in order to obtain the total number of influenza cases per year in Brazil. The parameters we used, detailed in Table S3, are assumed to be constant over the analysis period. Influenza incidences from 2010 to 2017, obtained thanks to this methodology, are shown in Fig. 1. As our epidemiological model requires weekly incidence data, we extracted the weekly number of influenza positive samples reported to WHO FluNet in Brazil over the studied period [20]. We synchronized the peaks occurring during each season (average lag of 5 weeks) and then computed the average number of cases for each week to obtain an estimated epidemic profile representative of a typical influenza season in Brazil (Figure S1). We then applied this “typical” epidemic profile to the yearly incidence per age-group previously estimated to obtain weekly incidence from yearly incidence.

### Vaccination

Vaccination rates for the period 2010–2017 (Table S2) were obtained from the Information System of the Brazilian National Immunization program [26] and were used for the model calibration process. For the analysis, we applied the coverage rates observed in 2017 for all age-groups as it is more likely to correspond to current and future coverage rates in the country. We varied the coverage rate observed in the 6 m-5yo age group from 50 to 100% in the sensitivity analysis.

### Epidemiological model calibration

Probabilities of infection were estimated for each influenza A subtypes and B lineages and estimated two by two (the two influenza A and the two influenza B). Estimations were performed sequentially for each year, on weekly influenza incidence, following the method described in Crépey et al. [15]. This method ensures that the level of immunity in the population for a given year depends on the influenza epidemic dynamics observed the previous years. We improved the calibration process developed in Crépey et al. [15] by estimating an age-based susceptibility vector allowing to reproduce more accurately influenza incidence observed in age-groups. As influenza epidemics in Brazil do not start at the same time depending on the latitude [27], epidemic curve shapes at the national level are difficult to fit with a model simulating a single epidemic on an single population. Consequently, in addition to matching the weekly incidence, the model was forced to replicate the yearly incidence in order to ensure that the model outcomes were consistent with the number of influenza cases observed in Brazil.

### Health outcomes data

All health outcomes data are detailed in supplementary material (Table S3 and S4). Probabilities of outpatient visits in case of influenza are taken from Prosser et al. [28] and Molinari et al. [29]. Although we obtained the number of hospitalizations for influenza in Brazil, the number of influenza cases in Brazil is not directly available and we could not document the probabilities of hospitalization per case (case/hospitalization ratio) in the Brazilian context, hence probabilities of hospitalization in case of influenza are taken from Reed et al. [30] in the US context. Probabilities of death in case of symptomatic influenza for each age-groups were extracted from published CDC estimates and averaged over the seasons 2012–2013 to 2016–2017 [31]. Influenza test sensitivity and probabilities of being tested were also taken from a study based in the US [24]. We used the estimated population size by age group and the life expectancy estimates over the period for Brazil from the Geographic and Statistic Brazilian Institute [32]. Due to the lack of utility estimates specific to Brazil, we used data from the US for quality adjusted life years lost and utility loss due to influenza and its consequences [28, 33].

### Costs data

All costs used in the model are detailed in supplementary material (Table S5).

#### Medical costs and indirect costs

Outpatient cost and medical cost of deaths are taken from SIGTAP [34], while hospitalization costs were provided by DATASUS [35] and averaged over 2010–2017. Treatment cost considers only the public cost of antiviral treatment in Brazil as we did not consider over-the-counter medication for simplification reasons, and since in many cases these represent out-of-pocket expenses. Private costs are detailed in the supplementary material. Productivity losses were estimated based on daily wages in Brazil [32] and inflated to the year 2017. The number of workdays lost due to influenza are estimated according to Molinari et al. [29]. Productivity losses due to mortality are estimated by computing the loss in earnings for the life years lost.

#### Vaccination cost

For the cost of a dose of TIV (0.5 ml), we consider the price published by the Brazilian government of R$15.14 [36]. For the QIV price, we considered R$33.89 (0,5 mL) which is the maximum manufacturer price without taxes published by Brazilian Medicines Market Regulation Chamber (CMED) [37]. We considered that the cost of a pediatric dose (0.25 ml) was half the cost of the adult dose. We did not account for administration cost in the analysis, as it would not make a difference since TIV and QIV are assumed to have the same coverage rate.

### Vaccine efficacy data

We considered vaccine efficacy per age and per influenza A subtypes (A/H1N1, A/H3N2) and B lineages (B Victoria, B Yamagata) as described in Crépey et al. [15] and shown in Table S6. Regarding cross-immunity between B lineages, we considered that a mismatched vaccine conferred 70% of the matched efficacy [38]. This cross-immunity estimate was varied in a dedicated sensitivity analysis.

### Scenarios analysis

A post-pandemic retrospective time horizon of 8 years from January 2010 to December 2017 was used in the calibration process in order to account for fluctuations in influenza incidence, influenza B circulation, and vaccine mismatch between seasons. However, we decided to not consider the year 2010 in our vaccine impact analysis to avoid the risk of biasing our results with the immediate aftermath of the 2009 pandemic. A high influenza A monovalent vaccination in 2009 may have triggered a proportionally higher influenza B circulation the following year (Table S1), which would have artificially favored the QIV strategy. To assess the vaccine impact, we used the 2011–2017 period and presented the averaged results of the 7-year period. The pandemic year 2009 was not considered as well for not being representative of the current epidemiological context. Incremental cost effectiveness ratios (ICER) were computed by dividing the net incremental cost of the strategy, compared to the baseline, by the net difference in QALYs or LYs. We considered one time the gross domestic product per capita (GDP) as a threshold for a “highly cost-effective” strategy (R$32,747) [39] and three times the GDP (R$98,241) for a cost-effective strategy. The public payer perspective was considered (SUS) but we also presented the societal perspective (including the private direct costs). According to Brazilian economic evaluations guidelines [40], all costs and health outcomes were discounted at a rate of 5%.

### Sensitivity analysis

We performed sensitivity analysis on vaccination coverage of the pediatric population, cross-protection between B-lineages (from no cross-immunity to 90% of the matched efficacy) and on influenza B circulation (from 20 to 40%). We also provide a deterministic sensitivity analysis on probabilities and costs of health outcomes in order to identify the main drivers of our results. Finally, to account for uncertainty in probabilities of outcomes and costs, and assess the robustness of our results, we performed an uncertainty analysis, also called probabilistic sensitivity analysis, on all costs and probabilities of outcome, whose range and probability distribution are given in Tables S3, S4 and S5. From this analysis, we provide a cost-effectiveness acceptability curve where 1000 simulations with different combinations of parameters are displayed.

## Results

Figure 1 shows the results of the epidemiological model calibration. Influenza incidences estimated by the model reflect relatively closely the observed incidence, with variations possibly due to the nature of influenza epidemics in Brazil. These simulated incidences are consistent with WHO estimates (between 5 and 10%) [1].

**Fig. 2:**
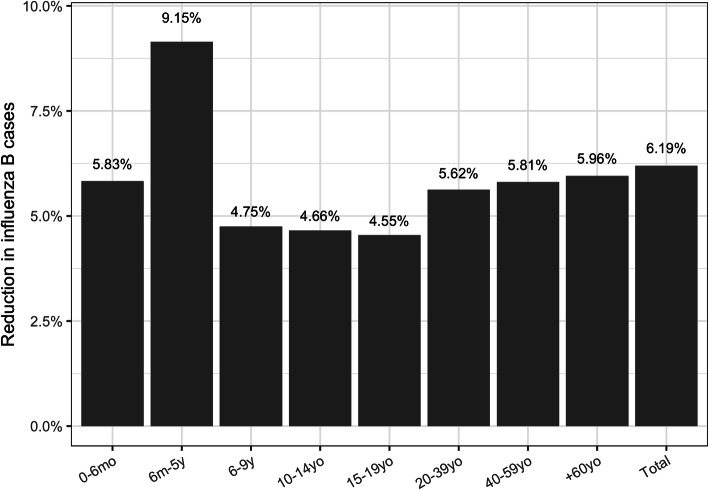
Impact by age-group of the switch to QIV on the reduction of symptomatic influenza B cases

Figure 2 shows the impact by age-group of the switch from TIV to QIV in the pediatric age-group. The targeted age-group would see a reduction up to 9.15% of the number of influenza B cases, while non-targeted groups would see a reduction up to 6% (> = 60yo) through indirect effects.

The public health and economic impact of switching from TIV to QIV in the pediatric age-group is detailed in Table 1. Our analysis shows that the switch to QIV would prevent ~ 406,600 symptomatic influenza B cases and ~ 400 deaths yearly, which could be translated into ~ 3400 life-years saved. Regarding costs, the averted productivity losses represent almost half of the cost of vaccination. Over the period 2011–2017, the switch to QIV in the pediatric population would have prevented 2.8 M symptomatic influenza B cases, 79,000 hospitalizations, and 2800 deaths. Single year impact of the pediatric switch to QIV is provided in Table S8 for each year of the analysis period.

**Table 1:** Outcomes, health effects and discounted costs for the baseline and QIV strategy

	Baseline	Scenario: QIV switch for 6 m-5yo	
		New situation	Difference
**Clinical influenza B outcomes (per season)**			
Total number of symptomatic cases	6,564,965	6,158,397	−406,569
Total number outpatient visits	2,341,626	2,188,848	− 152,778
Total number of hospitalizations	189,677	178,359	−11,318
Total number of deaths	6596	6203	− 393
**Health effects related to influenza B (per season)**			
Total QALYs lost because of influenza illness	61,646	57,902	− 3744
Total QALYs lost because of influenza-related deaths	47,861	45,304	− 2556
Total life-years lost because of influenza-related deaths	63,711	60,313	− 3398
**Discounted costs (R$)**			
Vaccination	687,307,209	863,977,370	176,670,161
Outpatient visit	92,744,270	87,410,202	−5,334,068
Hospitalized	196,767,867	186,295,892	−10,471,974
Death	20,417,936	19,332,171	−1,085,765
Productivity losses	1,351,536,346	1,278,078,007	− 73,458,340

Results are averaged over the 7 seasons considered. Only influenza B outcomes are considered since the switch to QIV only affects influenza B cases. Costs are discounted at 5% per year

Cost-effectiveness analysis are summarized in Table 2 and converted in US dollars in Table S7. The analysis shows that a QIV strategy for the pediatric age-group would be highly cost-effective with an ICER of R$26,798 per QALY or R$49,692 per LY saved (public payer perspective, discounted). Since most of prevented cost are societal, the strategies would even more cost-effective from a societal perspective.

**Table 2:** Public health and economic impact of the QIV strategy compared to TIV

	QIV strategy vs TIV strategy
**Incremental costs (R$)**	
Public payer direct costs	193,738,203
Public payer direct costs (discounted)	168,840,170
Direct costs (public & private) + societal costs	90,041,085
Direct costs (public & private) + societal costs (discounted)	86,320,015
**Incremental health outcomes**	
Life year saved	7762
Life year saved (discounted)	3398
QALY saved	10,484
QALY saved (discounted)	6301
**ICER (R$ per LY gained. Ref: current strategy)**	
Public payer perspective	24,960
Public payer perspective (discounted)	49,692
Societal perspective	11,600
Societal perspective (discounted)	25,405
**ICER (R$ per QALY gained, ref: current strategy)**	
Public payer perspective	18,480
Public payer perspective (discounted)	26,798
Societal perspective	8589
Societal perspective (discounted)	13,700

*ICER* Incremental cost-effectiveness ratio, *QALY* Quality-adjusted life-year

**Fig. 3:**
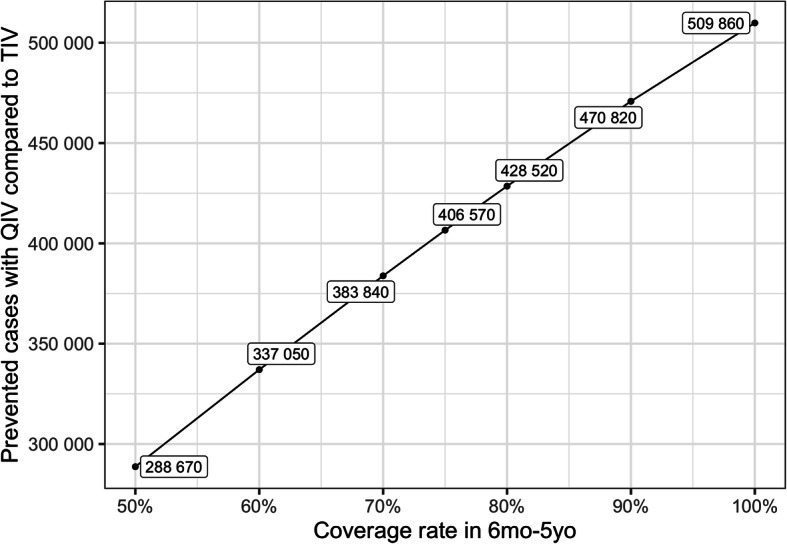
Sensitivity analysis on coverage rate

We also performed a sensitivity analysis on the coverage rate within the 6 m-5yo age-group. Figure 3 shows that varying the vaccination coverage from 50 to 100% in this age-group would increase the number of cases avoided from 288,670 to 509,860 yearly.

**Fig. 4:**
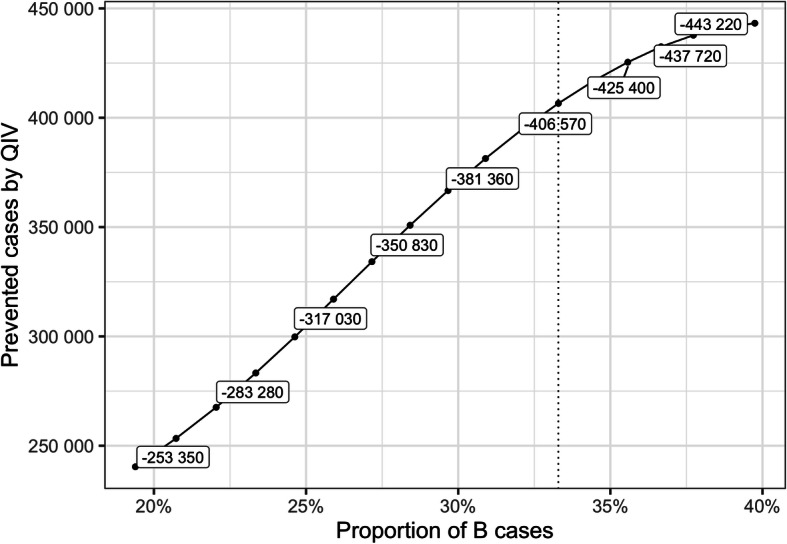
Sensitivity analysis on Influenza B circulation

In addition, we also explored the impact of influenza B circulation on our results (Fig. 4). On average over the period, we observe around 33% of Influenza B cases. Variations from 20 to 40% of this proportion would change the impact of QIV with a reduction in the number of cases ranging from 253,350 to 443,220 respectively.

**Fig. 5:**
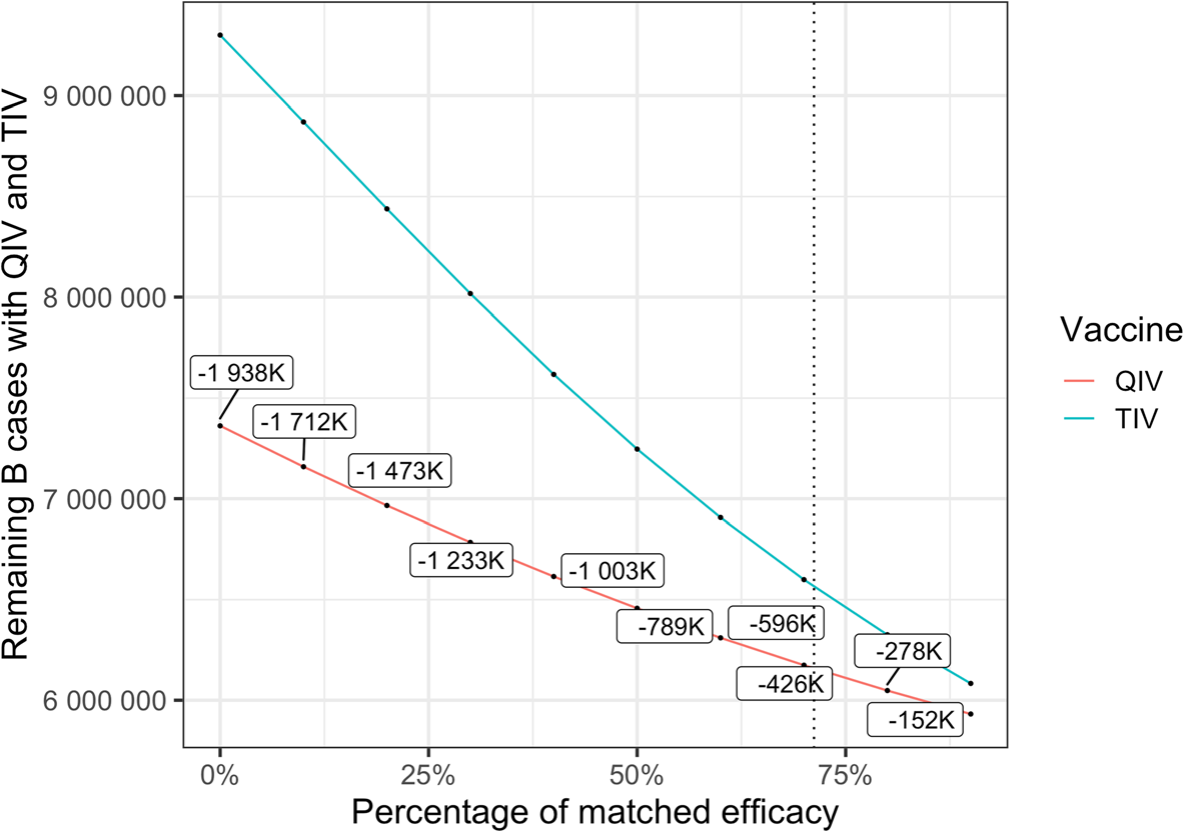
Sensitivity analysis on cross-immunity. The dotted line stands for the percentage of matched efficacy used in the model in case of TIV mismatch

A key driver in QIV impact is the level of cross-immunity considered in the analysis. Exploring the impact of this parameter (Fig. 5) on our results shows that the amplitude of the variations can be substantial. When no cross-immunity between Influenza B lineages are considered, the impact of QIV is at its maximum with almost 2 million influenza B cases that would not be prevented yearly with TIV, whereas 152,000 yearly cases would be prevented by QIV if the cross-immunity reaches 90%.

**Fig. 6:**
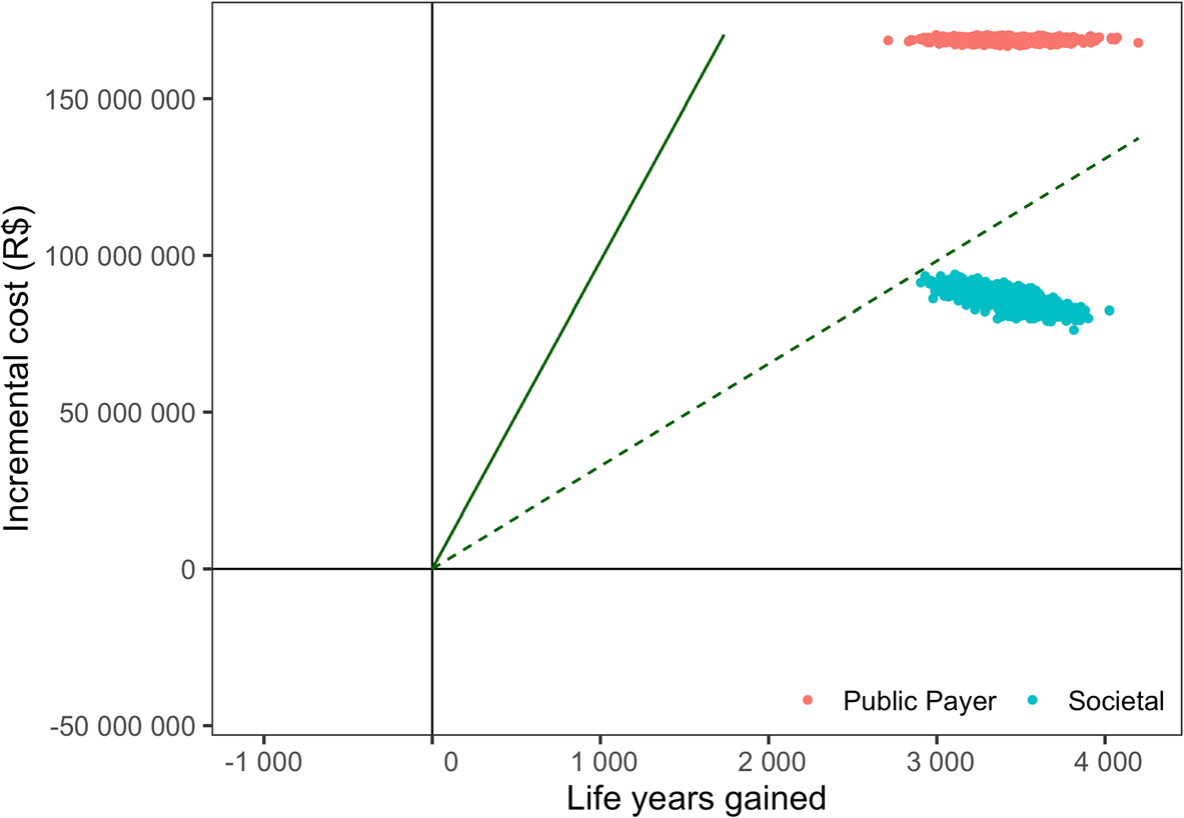
Probabilistic sensitivity analysis. Results of 1000 iterations of the model are displayed in a quadrant where the vertical axis stands for the incremental cost in R$ and the horizontal axis stands for life years gained. The dashed and plain green lines stand for cost-effectiveness ratios of one and three times the GDP per capita per life year gained, respectively

In order to assess the robustness of our results from an economic standpoint, we performed a probabilistic sensitivity analysis on the main economic inputs detailed in Table S4 and S5 and the main probabilities of outcomes detailed in Table S3. Results from this analysis (Fig. 6) show that switching to QIV remains cost-effective from a public payer perspective and highly cost-effective from a societal perspective when accounting for uncertainty in the parameters.

**Fig. 7:**
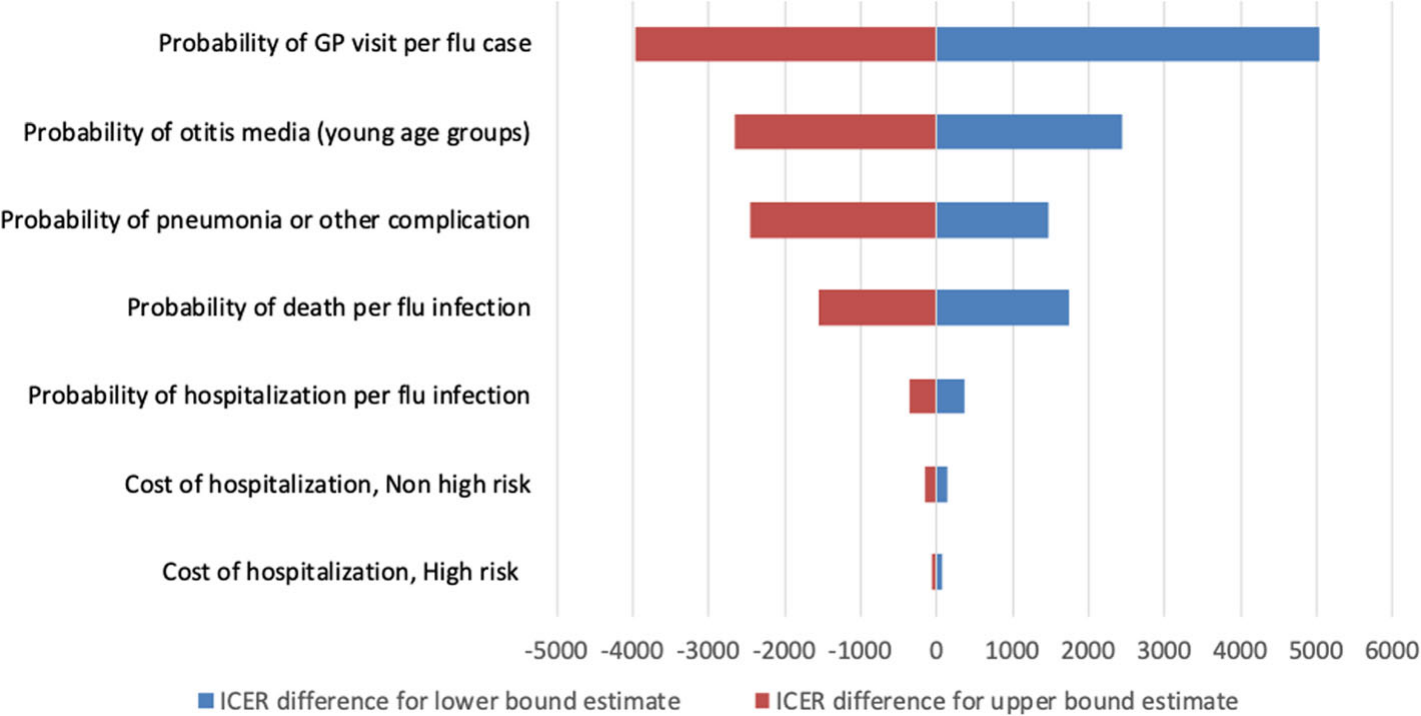
Tornado plot for the deterministic sensitivity analysis from the public payer perspective. Results are shown as differences from the incremental cost effectiveness ratio

We finally performed a deterministic sensitivity analysis whose results are shown as a tornado plot in Fig. 7. This analysis shows that the probability of visiting a GP for influenza has the highest impact on the cost effectiveness. However, in the most conservative scenario the ICER would increase by less than R$5000, and QIV would still remain cost-effective from a public payer perspective. We performed the same analysis from a societal perspective leading to the same conclusions (Figure S2).

## Discussion

Public health authorities may not be able to predict which B lineages will be circulating and which one will be dominant, particularly in a country crossing different climatic area like Brazil. A previous cost-effectiveness analysis already showed that switching from TIV to QIV would be a cost-effective strategy in Brazil, with ICERs ranging from R$20,000 to R$22,000 per QALY depending on the method used [13]. Our analysis reaches an ICER of similar magnitude when QIV is used specifically on the pediatric population but considering societal costs, indirect effect of vaccination, and various epidemiological context regarding influenza B circulation. Our results are in agreement with results from Jamotte et al. [12]. While they did not compute cost effectiveness ratios, they showed in their analysis that the pediatric age-group in Brazil would be the one benefitting the most from a better coverage rate and a more efficient vaccine. In the present analysis, we show that using QIV instead of TIV in the 6 m-5yo age-group is a cost-effective strategy from a third-party payer perspective, and a highly cost-effective one considering societal benefits. We conducted specific sensitivity analysis showing that the public health and economic impact of QIV depends on the coverage rate in the targeted population (Fig. 3), the proportion of influenza B circulating during the season (Fig. 4), and the level of cross-immunity between B lineages (Fig. 5). The variations highlighted in those analyses reinforce the rational of assessing the impact of QIV, compared to TIV, over multiple years in order to obtain the most relevant and contextualized results. The switch to QIV showed a strong public health impact, but the benefits are even more pronounced when the circulation of influenza B is high and when the cross-immunity between the two B lineages is poor. However, while the impact of QIV is reduced in case of TIV match with the circulating B strain, co-circulation of the two B lineages is very often observed (Table S1). Hence, TIV may remain sub-optimal against a large part of mismatched cases. Our simulated epidemics slightly differ from the one observed at the national level in Brazil since we are only able to simulate epidemics occurring at the same moment in different part of the country. Due to the large extent in latitude in the country, epidemic timing depends on the region, as described in the inter-tropical area and in large countries close to this area [4]. To simplify our analysis, we focused on the comparison between TIV and QIV and assumed that timing variations would impact the two strategies the same way, hence would be unlikely to qualitatively change our results. Our analysis relies on a previously published epidemiological model able to capture the dynamic aspects of influenza transmission. The model is able to qualitatively reproduce past influenza epidemics in Brazil but assumes inter-individual contacts similar to the one observed in the US population. We believe that this limitation may only reduce any indirect effect of influenza vaccination, and would not significantly change our results as both immunization strategies would be affected the same way.

Estimating the real burden of influenza is a difficult task in any country, particularly in the absence of a dedicated surveillance system outside of hospitals. A recent study has tried to estimate the influenza mortality burden in the Americas [41]. The researchers estimated a global death rate of 10 per 100,000 inhabitants for the PAHO region, and approximately 27,000 deaths yearly in the US alone. However, their results may well underestimate the real burden of the disease as the US CDC reported 79,000 deaths only for the season 2017–2018 [42], a country where universal influenza vaccination is the recommended strategy. A more recent assessment by the Global Burden of Disease initiative gave between 291,000 and 646,000 respiratory deaths globally [2], which increased the previous WHO estimation of 250,000 to 500,000 annual deaths. Indeed, their authors called for investigation of the still unknown burden of non-respiratory deaths due to influenza.

Given the lack of Brazilian data, our study largely used US estimates regarding influenza outcomes; hence our results are aligned with current US CDC estimations. While we acknowledge this use of international data as a limit of this analysis, we believe that any potential bias introduced by non-local data may, again, affect both immunization strategies the same way. In addition, our deterministic sensitivity analysis suggests that our results are relatively robust to variations in the probabilities of influenza severe outcomes (Figs. 6 and 7).

Potential uncertainties in unitary costs are also captured in our probabilistic sensitivity analysis, which does not display any contradictory claim (Fig. 7). The costs used in our economic analysis accounts for the costs funded by Federal Government, however the real cost for the hospital, −-and thus to the health system as a whole--, is much higher. For example influenza and pneumonia hospitalization cost could be three times more expensive than the cost estimated through DATASUS database [43]. Thus, the costs from the public payer perspective are likely to be significantly underestimated.

As noted by a recent systematic review of health economic evaluations of vaccines in Brazil [44], utility weights are not available for Brazil. Due to this lack of utility data specific to the Brazilian context, we presented ICERs per QALY and per LY, although the WHO cost-effectiveness threshold is only defined for ICER per QALY.

National Immunization Programs have the goal to maximize the public health impact of vaccination. Our analysis shows that both switching to QIV and achieving higher vaccination coverage would significantly contribute to this purpose.

## Conclusions

Based on the influenza epidemiological context in Brazil during the period 2011–2017, switching from TIV to QIV to protect children aged 6mo to 5yo could have a strong public health impact, while being a cost-effective strategy from a public payer perspective, even a highly cost-effective one from a societal perspective. Our analysis shows that improving coverage rates and improving vaccine effectiveness by using vaccines protecting against co-circulating B lineages are complementary strategies that could raise potential public health benefits for Brazil.

## Supplementary information

**Additional file 1 **: **Figure S1.** Annual influenza peaks in Brazil.

**Additional file 2 **: **Figure S2.** Tornado plot for the deterministic sensitivity analysis from the societal perspective.

**Additional file 3 **: **Table S1.** Proportions of influenza A/H1N1, A/H3N2, B Victoria, B Yamagata circulating for the period 2010–2017 in Brazil.

**Additional file 4 **: **Table S2.** Historical coverage rates per age-groups over the period 2010–2017 in Brazil.

**Additional file 5 **: **Table S3.** Health outcomes probabilities.

**Additional file 6 **: **Table S4.** Life expectancy and utility parameters.

**Additional file 7 **: **Table S5.** Cost parameters used in the economic model.

**Additional file 8 **: **Table S6.** Vaccine efficacy per strain and per age group.

**Additional file 9 **: **Table S7.** Public health and economic impact of the QIV strategy compared to TIV. Costs and ICER are given in 2017 US dollars.

**Additional file 10 **: **Table S8.** Single year impact of the switch from TIV to QIV in the pediatric population. In this analysis the model is run on each influenza season separately. The population in the QIV scenario and the basecase scenario starts each year with the same immune status, hence there is no additional build-up of naturally acquired immunity in the population vaccinated with TIV as it happens when simulations are performed over multiple years.

## Data Availability

Model code is available upon request. All the datasets (SINAN, WHO-Flunet, SUS/SIH) used and analysed during the current study are public and available from the corresponding author on reasonable request. All data generated during this study are available from the corresponding author on reasonable request.

## Abbreviations

ANS: Agência Nacional de Saúde Suplementar (Private National Health Agency)
CDC: Centers for Disease Control and Prevention
CMED: Câmara de Regulação do Mercado de Medicamentos (Brazilian Medicines Market Regulation Chamber)
DATASUS: Departamento de Informática do Sistema Único de Saúde
E: Exposed
GDP: Gross Domestic Product
ILI: Influenza-Like-Illness
I: Infectious
ICER: Incremental Cost Effectiveness Ratio
LY: Life Year
mL: Milliliter
MO: Month-Old
PAHO: Pan American Health Organization
QALY: Quality-Adjusted Life Year
QIV: Quadrivalent Influenza Vaccine
R: Recovered
S: Susceptible
SIGTAP: Sistema de Gerenciamento da Tabela de Procedimentos, Medicamentos e OPM do SUS
SINAN: Sistema de Informação de Agravos de Notificação (Notification Disease Information System)
SUS: Sistema Único de Saúde (Brazilian Public Health Care System)
TIV: Trivalent Influenza Vaccine
US: United States
V: Vaccinated
WHO: World Health Organization
YO: Year-Old
