# Supplementary figures and images for: Impact of quadrivalent influenza vaccines in Brazil: a cost-effectiveness analysis using an influenza transmission model
Source: BMC Public Health. 2020 Sep 9;20:1374. doi: 10.1186/s12889-020-09409-7 (PMC7487874; doi:10.1186/s12889-020-09409-7)

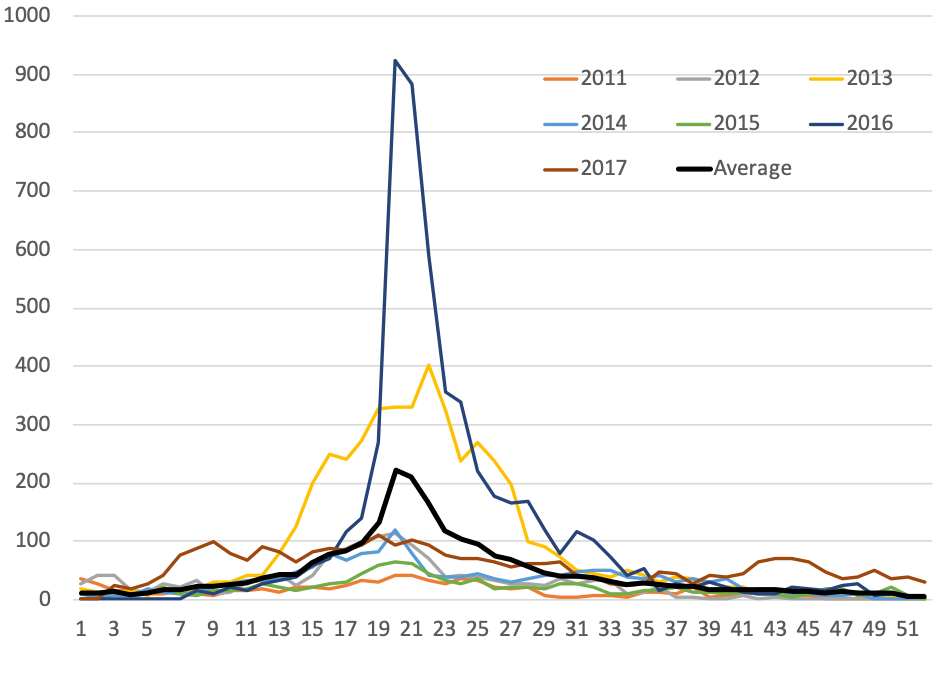

Supplement: Supplementary file 1 — Additional file 1 : Figure S1. Annual influenza peaks in Brazil. [file 12889_2020_9409_MOESM1_ESM.png]

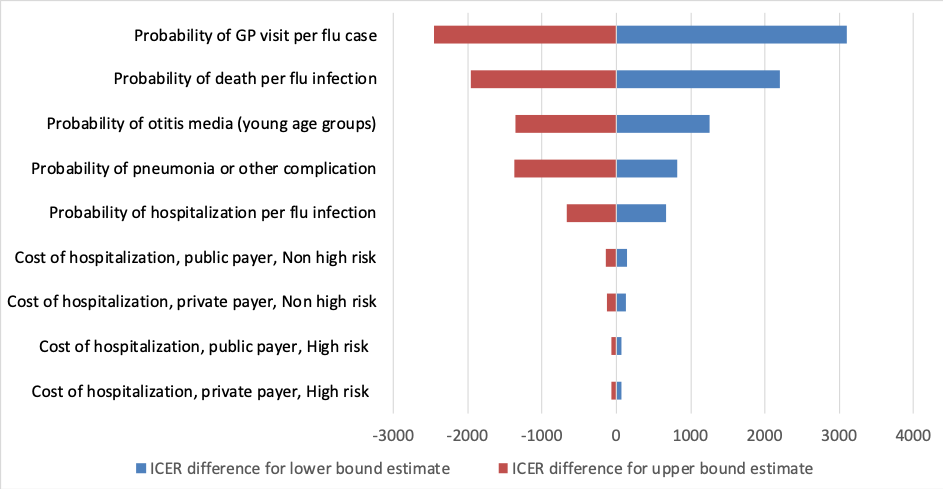

Supplement: Supplementary file 2 — Additional file 2 : Figure S2. Tornado plot for the deterministic sensitivity analysis from the societal perspective. [file 12889_2020_9409_MOESM2_ESM.png]
