# Supplementary material for: Impact of quadrivalent influenza vaccines in Brazil: a cost-effectiveness analysis using an influenza transmission model
Source: BMC Public Health. 2020 Sep 9;20:1374. doi: 10.1186/s12889-020-09409-7 (PMC7487874; doi:10.1186/s12889-020-09409-7)
Supplement: Supplementary file 3 — Additional file 3 : Table S1. Proportions of influenza A/H1N1, A/H3N2, B Victoria, B Yamagata circulating for the period 2010–2017 in Brazil. [file 12889_2020_9409_MOESM3_ESM.docx]

| **Year** | **A/H1N1** | **A/H3N2** | **B Victoria** | **B Yamagata** |
| --- | --- | --- | --- | --- |
| **2010** | 41.1% | 12.9% | 2.3% | 43.7% |
| **2011** | 19.7% | 46.1% | 34.2% | 0% |
| **2012** | 41.9% | 39.7% | 0.987% | 17.4% |
| **2013** | 39.5% | 21.6% | 36.6% | 2.23% |
| **2014** | 11.6% | 65.1% | 1.95% | 21.4% |
| **2015** | 8.39% | 55.0% | 3.81% | 32.8% |
| **2016** | 65.2% | 2.71% | 26.2% | 5.90% |
| **2017** | 0.6% | 60.9% | 6.63% | 31.9% |

Table S1: Proportions of influenza A/H1N1, A/H3N2, B Victoria, B Yamagata circulating for the period 2010-2017 in Brazil.
