# Supplementary material for: Impact of quadrivalent influenza vaccines in Brazil: a cost-effectiveness analysis using an influenza transmission model
Source: BMC Public Health. 2020 Sep 9;20:1374. doi: 10.1186/s12889-020-09409-7 (PMC7487874; doi:10.1186/s12889-020-09409-7)
Supplement: Supplementary file 4 — Additional file 4 : Table S2. Historical coverage rates per age-groups over the period 2010–2017 in Brazil. [file 12889_2020_9409_MOESM4_ESM.docx]

| **Age groups** | **2010** | **2011** | **2012** | **2013** | **2014** | **2015** | **2016** | **2017** |
| --- | --- | --- | --- | --- | --- | --- | --- | --- |
| **0-6m** | 0.00% | 0.00% | 0.00% | 0.00% | 0.00% | 0.00% | 0.00% | 0.00% |
| **6m-4yo** | 0.00% | 28.59% | 30.37% | 36.42% | 79.60% | 79.40% | 86.06% | 75.03% |
| **5-9yo** | 0.00% | 0.44% | 0.48% | 6.92% | 8.58% | 6.72% | 9.08% | 11.44% |
| **10-14yo** | 0.00% | 1.77% | 1.88% | 5.67% | 5.91% | 5.71% | 7.05% | 9.51% |
| **15-19yo** | 0.00% | 1.77% | 1.89% | 5.67% | 5.88% | 5.63% | 6.84% | 8.68% |
| **20-39yo** | 0.00% | 4.34% | 4.53% | 8.78% | 9.58% | 9.52% | 10.73% | 13.28% |
| **40-59yo** | 0.00% | 2.37% | 2.59% | 10.99% | 12.17% | 12.48% | 14.20% | 17.63% |
| **60+yo** | 79.07% | 80.46% | 79.75% | 83.23% | 78.21% | 77.89% | 81.34% | 73.19% |

Table S2: Historical coverage rates per age-groups over the period 2010-2017 in Brazil. These coverage rates are used only during the epidemiological model calibration phase.
