# Supplementary material for: Impact of quadrivalent influenza vaccines in Brazil: a cost-effectiveness analysis using an influenza transmission model
Source: BMC Public Health. 2020 Sep 9;20:1374. doi: 10.1186/s12889-020-09409-7 (PMC7487874; doi:10.1186/s12889-020-09409-7)
Supplement: Supplementary file 5 — Additional file 5 : Table S3. Health outcomes probabilities. [file 12889_2020_9409_MOESM5_ESM.docx]

| **Variables** | **Base case** | **Range** | | **Distribution** | **Reference** |
| --- | --- | --- | --- | --- | --- |
| Proportion of HR individuals |  |  | |  |  |
| 0 – 0,5 yo | 5.2% |  | |  | Molinari et al. 2007 |
| 0,5 – 5 yo | 5.2% |  | |  |  |
| 6 – 9 yo | 10.6% |  | |  |  |
| 10 – 14 yo | 10.6% |  | |  |  |
| 15 – 19 yo | 10.6% |  | |  |  |
| 20 – 39 yo | 24.4% |  | |  |  |
| 40 – 59 yo | 35.1% |  | |  |  |
| 60+ yo | 50.0% |  | |  |  |
| Probability of outpatient visit for flu infection | |  | |  |  |
| 0 – 0,5 yo | 0.50 | +/- 66% | | Beta | Prosser et al. 2011, Molinari et al. 2007  Note : twice for HR |
| 0,5 – 5 yo | 0.47 |  |  |  |  |
| 6 – 9 yo | 0.28 |  |  |  |  |
| 10 – 14 yo | 0.24 |  |  |  |  |
| 15 – 19 yo | 0.24 |  |  |  |  |
| 20 – 39 yo | 0.31 |  |  |  |  |
| 40 – 59 yo | 0.31 |  |  |  |  |
| 60+ yo | 0.62 |  |  |  |  |
| Probability of otitis media / influenza-related outpatient visit | | | | | |
| 0 – 0,5 yo | 0.63 | 0.33 | 0.80 | Beta | Prosser et al. 2011 |
| 0,5 – 5 yo | 0.5 | 0.27 | 0.80 |  |  |
| 6 – 9 yo | 0.23 | 0.05 | 0.50 |  |  |
| 10 – 14 yo | 0.15 | 0.01 | 0.40 |  |  |
| Probability of pneumonia or other complications / influenza-related outpatient visit | | | | | |
| 0-6 m | 0.2 | 0.04 | 0.5 | Beta | Prosser et al. 2011 |
| 6-59 m | 0.15 | 0.02 | 0.4 |  |  |
| 5-9 y | 0.11 | 0.02 | 0.3 |  |  |
| 10-14 y | 0.08 | 0.01 | 0.2 |  |  |
| Probability of hospitalization / flu case |  |  | |  |  |
| 0 – 0,5 yo | 0.007 | 0.00525 | 0.00875 | Beta | Reed et al. 2009 |
| 0,5 – 5 yo | 0.007 | 0.00525 | 0.00875 |  |  |
| 6 – 9 yo | 0.003 | 0.00203 | 0.00338 |  |  |
| 10 – 14 yo | 0.003 | 0.00203 | 0.00338 |  |  |
| 15 – 19 yo | 0.006 | 0.00420 | 0.00700 |  |  |
| 20 – 39 yo | 0.011 | 0.00795 | 0.01325 |  |  |
| 40 – 59 yo | 0.091 | 0.06825 | 0.11375 |  |  |
| 60+ yo | 0.091 | 0.06825 | 0.11375 |  |  |
| Probability of death / flu case |  |  | |  |  |
| 0 – 0,5 yo | 0.0000693 | 0.0000589 | 0.0000797 | Beta | Centers for Disease Control and Prevention, National Center for Immunization and Respiratory Diseases (NCIRD),  Averaged over 5 seasons (2012-2017), <https://www.cdc.gov/flu/about/burden/>  Note: Range is +/- 15% |
| 0,5 – 5 yo | 0.0000693 | 0.0000589 | 0.0000797 |  |  |
| 6 – 9 yo | 0.0000503 | 0.0000427 | 0.0000578 |  |  |
| 10 – 14 yo | 0.0000503 | 0.0000427 | 0.0000578 |  |  |
| 15 – 19 yo | 0.0001054 | 0.0000896 | 0.0001212 |  |  |
| 20 – 39 yo | 0.0001880 | 0.0001598 | 0.0002162 |  |  |
| 40 – 59 yo | 0.0003888 | 0.0003305 | 0.0004471 |  |  |
| 60+ yo | 0.0112037 | 0.0095231 | 0.0128842 |  |  |

**Table S3: Health outcomes probabilities**
