# Supplementary material for: Impact of quadrivalent influenza vaccines in Brazil: a cost-effectiveness analysis using an influenza transmission model
Source: BMC Public Health. 2020 Sep 9;20:1374. doi: 10.1186/s12889-020-09409-7 (PMC7487874; doi:10.1186/s12889-020-09409-7)
Supplement: Supplementary file 6 — Additional file 6 : Table S4. Life expectancy and utility parameters. [file 12889_2020_9409_MOESM6_ESM.docx]

| **Health effect** | **Base case** | **Range** | **Distribution** | **Reference** |
| --- | --- | --- | --- | --- |
| Baseline utility |  |  |  | GBD 2013. Global, regional, and national disability-adjusted life years (DALYs) for 306 diseases and injuries and healthy life expectancy (HALE) for 188 countries, 1990–2013: quantifying the epidemiological transition. |
| 0 – 0,5 yo | 0.967 |  |  |  |
| 0,5 – 5 yo | 0.975 |  |  |  |
| 6 – 9 yo | 0.953 |  |  |  |
| 10 – 14 yo | 0.931 |  |  |  |
| 15 – 19 yo | 0.919 |  |  |  |
| 20 – 39 yo | 0.897 |  |  |  |
| 40 – 59 yo | 0.851 |  |  |  |
| 60+ yo | 0.781 |  |  |  |
| QALY loss per outpatient influenza episode | |  |  |  |
| 0 – 19 yo | 0.005 | 0.002- 0.009 | Beta | Prosser et al. 2011 |
| 20 – 60+ yo | 0.0082 | 0.004672- 0.011728 | Beta | Bilcke et al. 2014 |
| QALY loss per episode of otitis media | |  |  |  |
| <18yo | 0.042 | 0.023-0.065 | Beta | Prosser et al. 2011 |
| QALY loss per episode of other influenza complications | |  |  |  |
| <18yo | 0.046 | 0.027-0.071 | Beta | Prosser et al. 2011 |
| QALY loss per inpatient influenza episode | |  |  |  |
| 0 – 19 yo | 0.076 | 0.054-0.1 | Beta | Prosser et al. 2011 |
| 20 – 60+ yo | 0.018 | 0.01-0.026 | Beta | Baguelin et al. 2015 |
| Life expectancy |  |  |  | IBGE, Diretoria de Pesquisas (DPE), Coordenação de População e Indicadores Sociais (COPIS) 2013 |
| 0 – 0,5 yo | 74.90 |  |  |  |
| 0,5 – 5 yo | 73.88 |  |  |  |
| 6 – 9 yo | 69.26 |  |  |  |
| 10 – 14 yo | 64.35 |  |  |  |
| 15 – 19 yo | 59.53 |  |  |  |
| 20 – 39 yo | 48.12 |  |  |  |
| 40 – 59 yo | 30.49 |  |  |  |
| 60+ yo | 14.24 |  |  |  |
| Life expectancy (discounted) |  |  |  |  |
| 0 – 0,5 yo | 19.53 |  |  |  |
| 0,5 – 5 yo | 19.50 |  |  |  |
| 6 – 9 yo | 19.37 |  |  |  |
| 10 – 14 yo | 19.20 |  |  |  |
| 15 – 19 yo | 18.98 |  |  |  |
| 20 – 39 yo | 18.13 |  |  |  |
| 40 – 59 yo | 15.52 |  |  |  |
| 60+ yo | 9.96 |  |  |  |
| Quality-adjusted life expectancy |  |  |  |  |
| 0 – 0,5 yo | 72.45 |  |  |  |
| 0,5 – 5 yo | 72.00 |  |  |  |
| 6 – 9 yo | 66.00 |  |  |  |
| 10 – 14 yo | 59.91 |  |  |  |
| 15 – 19 yo | 54.68 |  |  |  |
| 20 – 39 yo | 43.20 |  |  |  |
| 40 – 59 yo | 26.02 |  |  |  |
| 60+ yo | 11.00 |  |  |  |
| Quality-adjusted life expectancy (discounted) |  |  |  |  |
| 0 – 0,5 yo | 19.47 |  |  |  |
| 0,5 – 5 yo | 19.45 |  |  |  |
| 6 – 9 yo | 19.26 |  |  |  |
| 10 – 14 yo | 19.00 |  |  |  |
| 15 – 19 yo | 18.70 |  |  |  |
| 20 – 39 yo | 17.62 |  |  |  |
| 40 – 59 yo | 14.41 |  |  |  |
| 60+ yo | 8.25 |  |  |  |

**Table S4: Life expectancy and utility parameters**
