# Supplementary material for: Impact of quadrivalent influenza vaccines in Brazil: a cost-effectiveness analysis using an influenza transmission model
Source: BMC Public Health. 2020 Sep 9;20:1374. doi: 10.1186/s12889-020-09409-7 (PMC7487874; doi:10.1186/s12889-020-09409-7)
Supplement: Supplementary file 7 — Additional file 7 : Table S5. Cost parameters used in the economic model. [file 12889_2020_9409_MOESM7_ESM.docx]

| **Costs parameters** | **Base case** | **Range** | **Distribution** | **Reference** |
| --- | --- | --- | --- | --- |
| **Outpatient visit without complication** |  |  |  | Cost from public payer perspective (SUS) from DATASUS database.  Costs from private payer perspective have been calculated considering a percentage difference between public and private hospitalization costs found in the literature:   - +280% in a dengue study (Viera 2014) - +539% to 866% in a pneumo study (Neto 2011). - +160% in a dengue study (Martelli 2015)   Decision to use the middle value: +280% |
| All ages | R$ 10.00 | +/-25% | Gamma |  |
| All ages | R$ 58.16 | +/-25% | Gamma |  |
| **Outpatient visit pneumonia or other complications** | |  |  |  |
| Paediatric | R$ 36.67 | +/-25% | Gamma |  |
| Paediatric | R$ 248.76 | +/-25% | Gamma |  |
| Adult | R$ 59.08 | +/-25% | Gamma |  |
| Adult | R$ 299.32 | +/-25% | Gamma |  |
| **Hospitalization (public part)** |  |  |  |  |
| 0 – 0.5 yo | R$ 776.31 | +/-25% | Gamma |  |
| 0.5 – 5 yo | R$ 652.09 | +/-25% | Gamma |  |
| 6 – 9 yo | R$ 652.55 | +/-25% | Gamma |  |
| 10 – 14 yo | R$ 652.55 | +/-25% | Gamma |  |
| 15 – 19 yo | R$ 732.83 | +/-25% | Gamma |  |
| 20 – 39 yo | R$ 732.83 | +/-25% | Gamma |  |
| 40 – 59 yo | R$ 832.36 | +/-25% | Gamma |  |
| 60+ yo | R$ 816.61 | +/-25% | Gamma |  |
| **Hospitalization (private part)** |  |  |  |  |
| 0 – 0.5 yo | R$ 2 174 | +/-25% | Gamma |  |
| 0.5 – 5 yo | R$ 1 826 | +/-25% | Gamma |  |
| 6 – 9 yo | R$ 1 827 | +/-25% | Gamma |  |
| 10 – 14 yo | R$ 1 827 | +/-25% | Gamma |  |
| 15 – 19 yo | R$ 2 052 | +/-25% | Gamma |  |
| 20 – 39 yo | R$ 2 052 | +/-25% | Gamma |  |
| 40 – 59 yo | R$ 2 331 | +/-25% | Gamma |  |
| 60+ yo | R$ 2 286 | +/-25% | Gamma |  |
| **Medical cost per death (public part)** |  |  |  |  |
| 0 – 0.5 yo | R$ 1 740 | +/-25% | Gamma |  |
| 0.5 – 5 yo | R$ 1 740 | +/-25% | Gamma |  |
| 6 – 9 yo | R$ 1 740 | +/-25% | Gamma |  |
| 10 – 14 yo | R$ 1 740 | +/-25% | Gamma |  |
| 15 – 19 yo | R$ 1 833 | +/-25% | Gamma |  |
| 20 – 39 yo | R$ 1 833 | +/-25% | Gamma |  |
| 40 – 59 yo | R$ 2 308 | +/-25% | Gamma |  |
| 60+ yo | R$ 2 475 | +/-25% | Gamma |  |
| **Medical cost per death (private part)** |  |  |  |  |
| 0 – 0.5 yo | R$ 4 872 | +/-25% | Gamma |  |
| 0.5 – 5 yo | R$ 4 872 | +/-25% | Gamma |  |
| 6 – 9 yo | R$ 4 872 | +/-25% | Gamma |  |
| 10 – 14 yo | R$ 4 872 | +/-25% | Gamma |  |
| 15 – 19 yo | R$ 5 132 | +/-25% | Gamma |  |
| 20 – 39 yo | R$ 5 132 | +/-25% | Gamma |  |
| 40 – 59 yo | R$ 6 464 | +/-25% | Gamma |  |
| 60+ yo | R$ 6 932 | +/-25% | Gamma |  |
| **Lost workdays: Non-medically attended (days)** |  |  |  |  |
| 0 - 9 yo | 1 |  |  | Molinari et al 2007 |
| 10 – 59 yo | 0.5 |  |  |  |
| 60+ yo | 1 |  |  |  |
| **Lost workdays: Outpatient visit (N days)** |  |  |  |  |
| 0 – 0.5 yo | 1 |  |  |  |
| 0.5 – 5 yo | 1 |  |  |  |
| 6 – 9 yo | 1 |  |  |  |
| 10 – 14 yo | 1 |  |  |  |
| 15 – 19 yo | 1 |  |  |  |
| 20 – 39 yo | 1 |  |  |  |
| 40 – 59 yo | 2 |  |  |  |
| 60+ yo | 3 |  |  |  |
| **Lost workdays: HR Outpatient visit (N days)** |  |  |  |  |
| 0 – 0.5 yo | 6 |  |  |  |
| 0.5 – 5 yo | 6 |  |  |  |
| 6 – 9 yo | 4 |  |  |  |
| 10 – 14 yo | 4 |  |  |  |
| 15 – 19 yo | 4 |  |  |  |
| 20 – 39 yo | 2 |  |  |  |
| 40 – 59 yo | 4 |  |  |  |
| 60+ yo | 7 |  |  |  |
| **Lost workdays: Hospitalization (N days)** |  |  |  |  |
| 0 – 0.5 yo | 3.78 |  |  | Non high-risk: Ministério da Saúde - Sistema de Informações Hospitalares do SUS (SIH/SUS). Average Janu 2008- oct 2017 (total number of days divided by the total number of hospitalized cases ).  High risk: Molinari 2007 since the number of hospitalization days due to influenza for high risk people does not exist in DATASUS.  Note that considering the lenght of hospital stay for productivity lost is a conservative assumptions since hospitalized patients can stay some days at home after their hospitalization before going back to the office. |
| 0.5 – 5 yo | 3.78 |  |  |  |
| 6 – 9 yo | 3.24 |  |  |  |
| 10 – 14 yo | 3.24 |  |  |  |
| 15 – 19 yo | 3.24 |  |  |  |
| 20 – 39 yo | 3.74 |  |  |  |
| 40 – 59 yo | 4.61 |  |  |  |
| 60+ yo | 5.13 |  |  |  |
| **Lost workdays: HR Hospitalization (N days)** |  |  |  |  |
| 0 – 0.5 yo | 31 |  |  |  |
| 0.5 – 5 yo | 31 |  |  |  |
| 6 – 9 yo | 23 |  |  |  |
| 10 – 14 yo | 23 |  |  |  |
| 15 – 19 yo | 23 |  |  |  |
| 20 – 39 yo | 21 |  |  |  |
| 40 – 59 yo | 24 |  |  |  |
| 60+ yo | 18 |  |  |  |
| **Daily earnings for productivity losses** |  |  |  | Banco Central do Brasil, Calculadora do cidadão,  Value from 2015, therefore inflation rate considered to have value for 2017, It is considered that a caregiver is necessary for sick children. The earnings losses for caregivers is assumed to be the mean income of the population between 25 and 54 |
| 0 – 14 yo (parents) | R$52 |  |  |  |
| 15 – 19 yo | R$30 |  |  |  |
| 20 – 39 yo | R$40 |  |  |  |
| 40 – 59 yo | R$59 |  |  |  |
| 60+ yo | R$38 |  |  |  |
| **Vaccine price** |  |  |  |  |
| TIV | R$15.14 |  |  | (see text) |
| QIV | R$33.89 |  |  |  |

**Table S5: Cost parameters used in the economic model**
