# Supplementary material for: Impact of quadrivalent influenza vaccines in Brazil: a cost-effectiveness analysis using an influenza transmission model
Source: BMC Public Health. 2020 Sep 9;20:1374. doi: 10.1186/s12889-020-09409-7 (PMC7487874; doi:10.1186/s12889-020-09409-7)
Supplement: Supplementary file 8 — Additional file 8 : Table S6. Vaccine efficacy per strain and per age group. [file 12889_2020_9409_MOESM8_ESM.docx]

| **Variable** | **Base case** | **Reference** |
| --- | --- | --- |
| *Vaccine efficacy per strain* |  | DiazGranados et al. Vaccine 2012 and CDC unpublished data |
| AH1N1 |  |  |
| 0 - 0.5 y | 0 |  |
| 0.5 - 5 y | 0.5085 |  |
| 5 - 10 y | 0.473 |  |
| 10 - 15 y | 0.41 |  |
| 15 - 20 y | 0.41 |  |
| 20 - 40 y | 0.4165 |  |
| 40 - 60 y | 0.6665 |  |
| 60 - 100 y | 0.5 |  |
| AH3N2 |  |  |
| 0 - 0.5 y | 0 |  |
| 0.5 - 5 y | 0.5085 |  |
| 5 - 10 y | 0.473 |  |
| 10 - 15 y | 0.41 |  |
| 15 - 20 y | 0.41 |  |
| 20 - 40 y | 0.4165 |  |
| 40 - 60 y | 0.6665 |  |
| 60 - 100 y | 0.5 |  |
| B Victoria |  |  |
| 0 - 0.5 y | 0 |  |
| 0.5 - 5 y | 0.6102 |  |
| 5 - 10 y | 0.5676 |  |
| 10 - 15 y | 0.492 |  |
| 15 - 20 y | 0.492 |  |
| 20 - 40 y | 0.4998 |  |
| 40 - 60 y | 0.7998 |  |
| 60 - 100 y | 0.6 |  |
| B Yamagata |  |  |
| 0 - 0.5 y | 0 |  |
| 0.5 - 5 y | 0.6102 |  |
| 5 - 10 y | 0.5676 |  |
| 10 - 15 y | 0.492 |  |
| 15 - 20 y | 0.492 |  |
| 20 - 40 y | 0.4998 |  |
| 40 - 60 y | 0.7998 |  |
| 60 - 100 y | 0.6 |  |
