# Supplementary material for: Impact of quadrivalent influenza vaccines in Brazil: a cost-effectiveness analysis using an influenza transmission model
Source: BMC Public Health. 2020 Sep 9;20:1374. doi: 10.1186/s12889-020-09409-7 (PMC7487874; doi:10.1186/s12889-020-09409-7)
Supplement: Supplementary file 9 — Additional file 9 : Table S7. Public health and economic impact of the QIV strategy compared to TIV. Costs and ICER are given in 2017 US dollars. [file 12889_2020_9409_MOESM9_ESM.docx]

|  | **QIV strategy vs TIV strategy** |
| --- | --- |
| **Incremental costs (US$)** |  |
| Public payer direct costs | 58 121 461 |
| Public payer direct costs (discounted) | 50 652 051 |
| Direct costs (public & private) + societal costs | 27 012 325 |
| Direct costs (public & private) + societal costs (discounted) | 25 896 005 |
| **Incremental health outcomes** |  |
| Life year saved | 7 762 |
| Life year saved (discounted) | 3 398 |
| QALY saved | 10 484 |
| QALY saved (discounted) | 6 301 |
| **ICER (US$ per LY gained. ref: current strategy)** |  |
| Public payer perspective | 7 488 |
| Public payer perspective (discounted) | 14 908 |
| Societal perspective | 3 480 |
| Societal perspective (discounted) | 7 621 |
| **ICER (US$ per QALY gained, ref: current strategy)** |  |
| Public payer perspective | 5 543 |
| Public payer perspective (discounted) | 8 040 |
| Societal perspective | 2 576 |
| Societal perspective (discounted) | 4 110 |
| ICER: incremental cost-effectiveness ratio; QALY: quality-adjusted life-year. | |

Table S7: Public health and economic impact of the QIV strategy compared to TIV. Costs and ICER are given in 2017 US dollars with a conversion rate of US$ 1 = R$ 0.30 (source: xe.com conversion rate averaged over the year 2017).
