# Supplementary material for: Impact of quadrivalent influenza vaccines in Brazil: a cost-effectiveness analysis using an influenza transmission model
Source: BMC Public Health. 2020 Sep 9;20:1374. doi: 10.1186/s12889-020-09409-7 (PMC7487874; doi:10.1186/s12889-020-09409-7)
Supplement: Supplementary file 10 — Additional file 10 : Table S8. Single year impact of the switch from TIV to QIV in the pediatric population. In this analysis the model is run on each influenza season separately. The population in the QIV scenario and the basecase scenario starts each year with the same immune status, hence there is no additional build-up of naturally acquired immunity in the population vaccinated with TIV as it happens when simulations are performed over multiple years. [file 12889_2020_9409_MOESM10_ESM.docx]

|  | **Absolute difference with QIV** | | **Relative difference with QIV** | |
| --- | --- | --- | --- | --- |
| **Year** | **Cases (cumulative)** | **Cases (yearly)** | **Rate (per 100 000)** | **Cases (%)** |
| 2011 | 0 | 0 | 0 | 0 |
| 2012 | -2 372 100 | -2 372 100 | -1 367 | -10.16 |
| 2013 | -3 853 700 | -1 481 600 | -854 | -6.12 |
| 2014 | -4 044 800 | -191 100 | -110 | -0.81 |
| 2015 | -4 368 000 | -323 200 | -186 | -1.4 |
| 2016 | -4 480 200 | -112 200 | -65 | -0.5 |
| 2017 | -5 196 500 | -716 300 | -413 | -5.13 |
| **Average** |  | -742 400 | -428 |  |
| **Total** |  | -5 196 500 | -2 995 | -3.81 |

**Table S8:** Single year impact of the switch from TIV to QIV in the pediatric population. In this analysis the model is run on each influenza season separately. The population in the QIV scenario and the basecase scenario starts each year with the same immune status, hence there is no additional build-up of naturally acquired immunity in the population vaccinated with TIV as it happens when simulations are performed over multiple years.
